# A Water-Soluble Epoxy-Based Green Crosslinking System for Stabilizing PVA Nanofibers

**DOI:** 10.3390/molecules27134177

**Published:** 2022-06-29

**Authors:** Yujian Zhang, Kuanjun Fang, Wei Wang, Haitao Niu

**Affiliations:** 1 State Key Laboratory for Biofibers and Eco-Textiles, Collaborative Innovation Centre for Eco-Textiles of Shandong Province, College of Textiles & Clothing, Qingdao University, 308 Ningxia Road, Qingdao 266071, China; jadedchang@163.com (Y.Z.); wangviyeah@163.com (W.W.); 2 Jiangsu New Vision Advanced Functional Fiber Innovation Center, Shengze Town, Suzhou 215228, China

**Keywords:** crosslinking, electrospinning, epoxy, polyvinyl alcohol, water resistance

## Abstract

With the ever-growing concern about environmental conservation, green production and water-based nanofibers have attracted more and more interest from both academic and industrial fields; nevertheless, the stabilization process of water-based nanofibers is primarily relying on the application of organic solvent-based crosslinking agents. In this work, we develop a green approach to fabricate water-resistant polyvinyl alcohol (PVA) nanofibers by using a water-based epoxy compound, N^1^, N^6^-bis(oxiran-2-ylmethyl) hexane-1,6-diamine (EH), as the crosslinker. This EH/sodium carbonate/sodium bicarbonate (CBS) solution system can break down large aggregates of PVA molecules into small ones and promote the uniform distribution of EH in the solution, resulting in the improved stability of crosslinked PVA nanofibers. We firstly report that the uniform dispersion of crosslinking agents in the electrospinning solution plays a vital role in improving the stability of spinning solutions and the water resistance of crosslinked PVA nanofibers by comparing crosslinking performances between water-based epoxy and conventional water-based blocked isocyanate (BI). This work could open up a novel strategy and green approach for the stabilization of water-based nanofibers.

## 1. Introduction

In the past few decades, electrospinning has become a prevalent method for preparing nanofibrous materials because of its ease of manufacturing, high efficiency and multiple functions. Attributed to their high volume-to-length ratio, large specific surface area, small pore size and high porosity, electrospun nanofibers have found applications in a wide range of fields, such as air filtration [1], water treatment [2], energy production [3], medical and bioengineering [4,5], environmental remediation [6], etc. Synthetic polymers, e.g., polyacrylonitrile [7], polyvinylidene fluoride [8], polyamide [9], nylon [10] and polyvinyl butyral [11], are generally used to make electrospun nanofibers because of their excellent spinnability, high purity, large molecular weight and outstanding performances. Compared with the above-mentioned organic solvent-soluble polymers, the manufacturing and application of water-soluble polymer nanofibers have many remarkable merits, e.g., no release of organic solvent, safe production and low cost. In addition, the existence of hydrophilic groups, such as hydroxyl, carboxyl and amide in these water-soluble polymers imparts them with outstanding properties, such as good adhesion, gelatinization and ease of functionalization. There are many water-soluble polymers suitable for electrospinning, including polyvinyl alcohol [12], poly (ethylene oxide) [13], polyvinyl pyrrolidone [14], sodium alginate [15], polyacrylic acid [16], etc., of which polyvinyl alcohol (PVA) is one of the most studied and broadly used water-soluble polymers.

PVA has many outstanding properties, e.g., good adhesion, oil resistance, degradability and biocompatibility, and as a result, it has been used for various applications such as electronic product packaging film [17], clothing and food packaging film [18], paper making [19], adhesives [20], fabric finishing agents [21], etc. Compared with PVA dense membranes, PVA nanofiber membranes have a large specific surface area, small pore size, large porosity and high permeability, which enables them to be used in diversified fields, e.g., filtration [1], biomedical materials [22], energy [23] and environment [6]. For practical applications, it is preferable and essential to stabilize PVA molecules by crosslinking hydroxyl groups with functional groups. Because the electrospinning process happens instantaneously with nanofibers produced in microseconds, many PVA crosslinking agents used in the traditional spinning technology may not be applicable. For example, some free radical reaction crosslinkers, such as N-(methoxymethyl)-acrylamide(MMAM), N-(n-butoxymethyl)-acrylamide(BMAM) or N-methylol-acrylamide(MAM), cannot be used to crosslink PVA nanofibers due to the complex and slow crosslinking process [24]. Organic solvent-soluble chemicals, e.g., formaldehyde [25], glutaraldehyde [26,27,28], epichlorohydrin [29], glycidyl methacrylate [30] and epoxy resin [31], are the most frequently used chemicals for crosslinking PVA macromolecules, with satisfactory performances. For instance, glutaraldehyde is currently one of the most-used crosslinking agents in the fiber industry for producing vinylon fibers, albeit they can be correlated to neurodegenerative disease, heart disease and some types of cancer. Because these crosslinking agents are toxic and can cause harm to the environment, environmental friendly water-based crosslinking agents, e.g., citric acid [32,33,34], tartaric acid [35], polyacrylic acid [12,36] and water-based blocked isocyanate [37], have become more and more employed. They may still face many obstacles in practice, including low crosslinking efficiency, acidic and corrosive solution and uneven mixing with PVA molecules due to the forms of long macromolecular chains or emulsions. The stability of spinning solutions and uniformity of electrospun nanofibers are important prerequisites for choosing crosslinkers. The homogeneous distribution of crosslinking agents in the PVA solution could have a substantial impact on the crosslinking efficiency; however, no relevant research has been reported.

In this research, we developed an environmentally friendly approach to crosslink PVA nanofibers with excellent water resistance, revealing the importance of homogeneous dispersion of crosslinking agents in PVA nanofibers on improving the fiber stability. The water-soluble epoxy compound N^1^, N^6^-bis(oxiran-2-ylmethyl) hexane-1,6-diamine (EH) was employed to crosslink PVA nanofibers, which was uniformly distributed in the spinning solution and nanofibers, and the produced nanofibers obtained stable crosslinked structure after heat treatment. The influences of processing conditions, e.g., solution homogeneity, heating temperature and time and addition of sodium carbonate/sodium bicarbonate (denoted as CBS) on the crosslinking efficiency have been studied. The crosslinking agent EH distributed uniformly within the nanofibers and produced highly stabilized PVA nanofibers. For comparison, water-based blocked isocyanate (BI) was unevenly distributed in the PVA nanofibers resulting in the relatively low water resistance of PVA nanofibers.

## 2. Materials and Methods

### 2.1. Materials

PVA (molecular weight = 75,000–180,000 g/mol, degree of hydrolysis = 87.0–89.0%) was obtained from Aladdin Reagent (Shanghai, China) Co., Ltd. Epoxy crosslinking agent EH (N^1^, N^6^-bis(oxiran-2-ylmethyl) hexane-1,6-diamine) (35% solid content) was purchased from Shaoxing Oucheng Chemical (Shaoxing, China) Co., Ltd. Water-based blocked isocyanate (35% solid content) was purchased from Guangzhou Yele New Material (Guangzhou, China) Co., Ltd. Sodium carbonate and sodium bicarbonate (CBS) were obtained from Sinopharm Chemical Reagent (Shanghai, China) Co., Ltd. All these reagents were of analytical grade. Ultrapure water was used throughout the experiments.

### 2.2. Preparation of Electrospinning Solutions

The PVA solution was prepared by dissolving 8 g PVA particles in 100 mL ultrapure water at 95 °C with mechanical stirring at 250 rpm for 12 h to obtain a homogeneous and transparent solution. The PVA/EH and PVA/EH/CBS solutions were prepared by mixing PVA solution with EH or EH/CBS at 25 °C with continuous stirring for 4 h, until a homogeneous viscous solution was obtained. The PVA to EH mass ratio was set as 1:1 and the CBS content in the solution was set as 0.2 mol/L constantly. The concentration of CBS in the solution is shown in Table 1. For comparison, a PVA/BI dispersion with mass ratio of 1:1 was also prepared by adding 1.6 g of BI dispersion to 20 mL of 80 g/L PVA solution by stirring at 25 °C for 4 h to obtain a homogeneous dispersion. 

### 2.3. Preparation of Nanofibers

PVA, PVA/EH and PVA/EH/CBS solutions and PVA/BI dispersion were electrospun in lab-made electrospinning equipment at the spinning temperature of 25 ± 3 °C and humidity of 25–30%. The obtained nanofibers were named as PVA nanofibers, PVA/EH nanofibers, PVA/EH/CBS nanofibers and PVA/BI nanofibers, respectively. Nanofibers were collected by a grounded steel drum wrapped with aluminium foils. The collected nanofibers were heated in an oven to induce crosslinking. The needle gauge size, flow rate of solution, collecting distance and the rotation rate of the collector were fixed at 21 gauge, 0.3 mL/h, 18 cm, 20 kV and 100 rpm/min, respectively.

### 2.4. Characterizations

The morphology of nanofibers was observed by field emission scanning electron microscopy (Regulus 8100, Tokyo, Japan). The elements in the fibers were analyzed by energy disperse spectroscopy (EDS) connected to FE-SEM (FEI Scios 2 HiVac). XPS analysis was carried out on Smartlab SE (Rigaku, Tokyo, Japan), and wide spectra in the range of 0–1100 eV and narrow spectra at high resolution for all elements were recorded. FTIR spectra of nanofiber membranes were recorded by a FTIR spectrometer (NICOLET iS10, Massachusetts, USA) at room temperature. The crystallization of nanofibers was investigated via an X-ray diffractometer (XRD, X’Pert-PRO, PANalytical) with Cu K_α_ radiation. The thermal properties of nanofibers were characterized by the thermogravimetric analysis (TGA, NETZSCH STA 449F3) with a heating/cooling rate of 20 °C/min in the nitrogen atmosphere with temperature range between 30 °C and 800 °C. The stability of the spinning solution was measured by a stability analyzer (Turbiscan Lab Expert, Formulation) at 25 °C with a scan frequency of 1 time/hour. The hydrodynamic diameter distribution was determined by a Litesizer 500 (DLS, Anton Paar Ltd., Graz, Austria) at 25 °C.

### 2.5. Water Resistance of Nanofibers

The nanofiber membranes were cut into a square of 4 cm^2^ and dried in a vacuum oven at 60 °C for 24 h to ensure complete drying. The dried nanofiber membranes (~0.03 g in weight) were immersed in 20 mL water at 25 °C for 120 min. After incubation, these samples were taken out and dried in an oven at 60 °C for 6 h. The weight loss of samples (W, %) was calculated using Equation (1)

(1) $$W\left(\%\right)=\frac{w_{i}{-w}_{f}}{w_{i}}\times 100\%$$

where w_i_ and w_f_ are the initial and final weights of samples, respectively. All the experiments were carried out in triplicate and their average weight loss was recorded.

## 3. Results and Discussion

**Figure 1:**
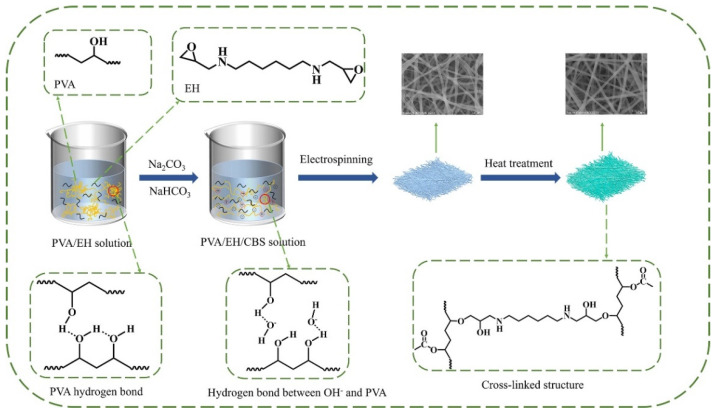
Schematic illustration of preparing PVA/EH/CBS nanofibers.

Figure 1 illustrates the preparation process of PVA/EH/CBS nanofibers. Firstly, a certain amount of CBS was added to the PVA solution to create an alkaline environment, then the crosslinking agent EH was added to the PVA/CBS solution. The prepared solution was electrospun in a lab-made electrospinning device, and the obtained nanofibers were heated to induce esterification reaction between epoxy groups of EH and hydroxyl groups of PVA to form a crosslinked structure. The PVA/EH/CBS nanofibers before and after heat treatment both had uniform fiber morphology with fiber diameters of 316.6 nm and 317.4 nm, respectively.

**Figure 2:**
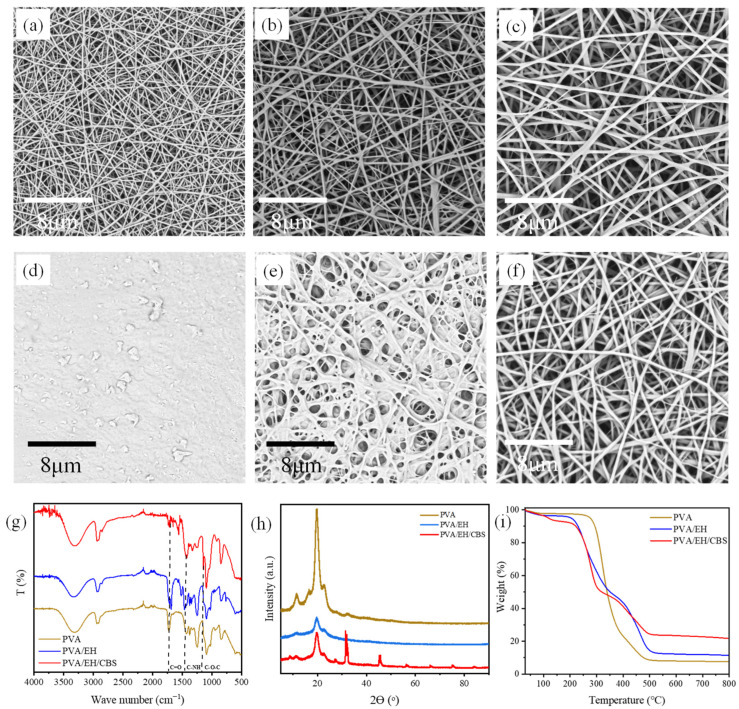
SEM images of (**a**,**d**) PVA nanofibers (**b**,**e**) PVA/EH nanofibers, (**c**,**f**) PVA/EH/CBS nanofibers before and after immersion in water, (**g**) FTIR spectra, (**h**) XRD patterns, (**i**) TG results of PVA, PVA/EH and PVA/EH/CBS nanofibers.

PVA nanofibers without heat treatment dissolved in water immediately. After heating at 160 °C for 10 min, the morphology of PVA nanofibers did not change significantly, as shown in Figure 2a. After being immersed in water, these heated PVA nanofibers dissolved, lost the fibrous morphology and formed a dense film, as shown in Figure 2d. This is because there are a large number of hydrophilic hydroxyl groups (87.0–89.0%) on PVA molecule chains. Although the high temperature treatment could induce etherification reaction between hydroxyl groups, only a small percentage of them took part in the reaction since etherification is very hard to proceed with in the absence of catalysts [38]. The unreacted hydroxyl groups on the molecule chains made PVA nanofibers soluble in water.

Figure 2b shows that uniform PVA/EH (mass ratio is 1:1) nanofibers were obtained. After being immersed in water for over 120 min, the PVA/EH nanofibers conserved their fiber morphology, but still experienced shrunk pore size and enlarged fiber diameter as shown in Figure 2e. Due to the low crosslinking degree, PVA/EH nanofibers can absorb a small amount of water and become sticky, accompanied by the noticeably declined mechanical strength when in contact with water. The crosslinked PVA/EH nanofibers have not met the fundamental requirements for practical applications yet.

Both the PVA aqueous solution and EH solution had acidic pH; however, it was reported that epoxy groups had high reaction rates with the hydroxyl group at alkaline conditions [39]. Therefore, it is reasonable to speculate that the reactivity of epoxy groups could be improved by adjusting the pH value of PVA/EH solutions to alkaline, resulting in PVA nanofibers with the improved crosslinked structure. Sodium carbonate and sodium bicarbonate were applied to adjust the pH value of PVA/EH solutions. Sodium carbonate had limited solubility in the PVA aqueous solution, whereas although sodium bicarbonate is a weak alkaline, the mixed solution of sodium carbonate/sodium bicarbonate had acceptable solubility and could adjust the pH value of PVA solution from 7.8 to 9.2 to catalyze esterification reaction. The obtained PVA/EH/CBS nanofibers had a uniform fibrous morphology and showed negligible morphology change after being immersed in water as shown in Figure 2c,f.

The FTIR spectra of PVA, PVA/EH and PVA/EH/CBS nanofibers are shown in Figure 2g. The absorption band at 1700–1750 cm^−1^ was assigned to the stretching vibration of carbonyl from the acetate groups present in the partially hydrolyzed PVA. There was a strong absorption peak at 1400–1480 cm^−1^ corresponding to C–N in the spectra of PVA/EH and PVA/EH/CBS nanofibers, and corresponding to C–N belonging to epoxy crosslinker EH, which proved crosslinker EH had been introduced into PVA nanofibers. In the PVA/EH/CBS nanofiber spectrum, there was a strong absorption peak at 1110–1190 cm^−1^ corresponding to C–O–C, which indicated that the etherification reaction between the epoxy group and the hydroxyl group occurred in PVA/EH/CBS nanofiber [39].

The crystal structure of nanofibers was studied by XRD as shown in Figure 2h. The diffraction peak at 19.82° was assigned to the (101) plane of the semi-crystalline structure of PVA with strong intermolecular hydrogen bonding [40]. The peak intensity of crosslinked nanofibers was expressively lower than that of pure PVA nanofibers, which could be attributed to fewer hydroxyl groups in the nanofibers. Hydroxyl groups could form intramolecular and intermolecular hydrogen bonds, thus improving the PVA chain arrangement and the crystallinity accordingly. In addition, the crosslinking agent EH distributed ubiquitously in the nanofibers interacting with PVA molecule chains and formed a crosslinked structure, which damaged the regularity of PVA molecule chains, resulting in decrease crystallinity. In the PVA/EH/CBS nanofiber diffraction pattern, there were two new crystallization peaks at 31.44° and 45.27°, corresponding to the diffraction plane of (021) and (041) of NaHCO_3_, respectively, and three new crystallization peaks at 56.21°, 65.97° and 75.05°, corresponding to the diffraction planes of (004), (301) and (222) of Na_2_CO_3_, respectively, which came from the precipitated Na_2_CO_3_ and NaHCO_3_ crystals in PVA nanofibers. 

The TGA results in Figure 2i shows the thermal behaviors of PVA, PVA/EH and PVA/EH/CBS nanofibers without thermal treatment. The thermal degradation of PVA nanofibers had two stages: removal of moisture and volatile contents at below 100 °C and decomposition at 300–500 °C. The results reveal that the PVA nanofibers had a small quantity of unevaporated water (about 7.28%), and the final residue mass was 7.68% at 800 °C. PVA/EH nanofibers had less water left inside (4.97%) and an increased residue mass of 11.39%. Due to the thermal decomposition of sodium bicarbonate, the weight loss of PVA/EH/CBS nanofibers at 200 °C was 9.44%, which was significantly more than that of PVA and PVA/EH nanofibers. The degradation temperature of PVA/EH and PVA/EH/CBS nanofibers was around 250 °C, and PVA nanofibers began to degrade at around 300 °C and were more stable. This is because the addition of EH can destroy the crystalline structure of PVA, as revealed in Figure 2h, which results in an early degradation of PVA/EH and PVA/EH/CBS nanofibers [41]. Since sodium carbonate is stable and does not decompose at 800 °C, the final residual amount of PVA/EH/CBS nanofibers was 21.74%, which was higher than that of PVA and PVA/EH nanofibers.

**Figure 3:**
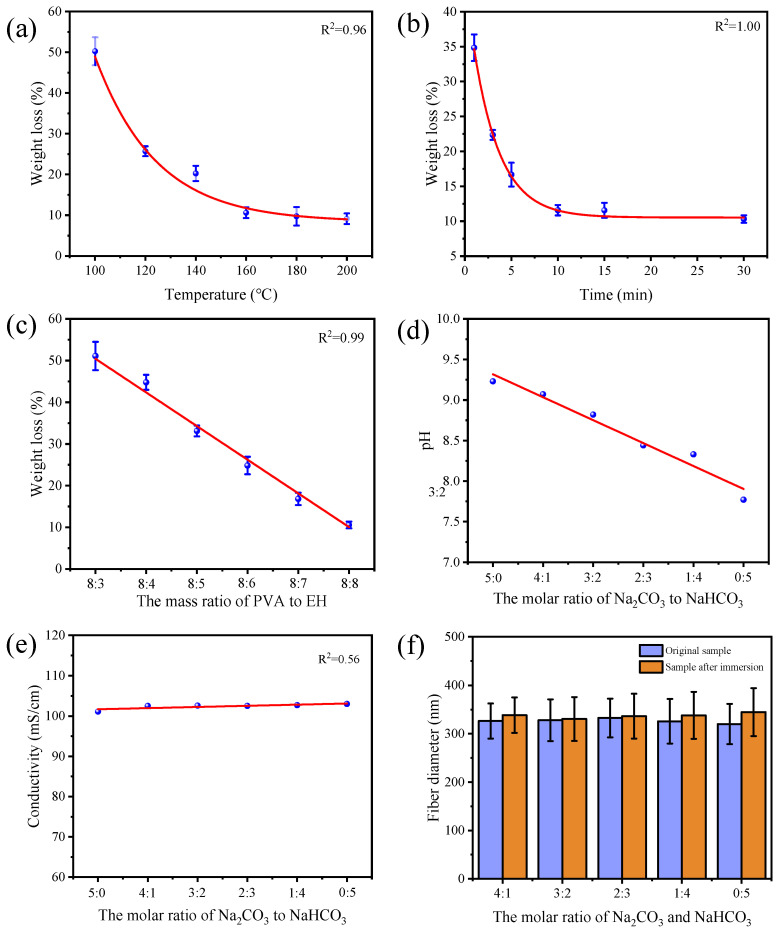
Influence of (**a**) heating temperature, (**b**) heating time and (**c**) PVA/EH mass ratio on weight loss of nanofibers immersed in water for 120 min (mass ratio of PVA and EH = 1:1, molar ratio of Na_2_CO_3_ and NaHCO_3_ = 3:2), (**d**) relationship between pH and Na_2_CO_3_/NaHCO3 molar ratio, (**e**) relationship between conductivity and Na_2_CO_3_/NaHCO_3_ molar ratio, (**f**) influence of molar ratio of Na_2_CO_3_/NaHCO_3_ on fiber diameter (mass ratio of PVA and EH = 1:1).

It was found that the temperature and time of heat treatment had obvious influences on the crosslinking reaction between epoxy and hydroxyl groups. The rising temperature and prolonged treatment both decreased the weight loss of PVA/EH/CBS nanofibers after being immersed in water, revealing an improved crosslinking degree of PVA nanofibers as shown in Figure 3a,b. When the treatment time was kept at 30 min, a heating temperature of lower than 140 °C could not induce enough crosslinking points among PVA molecules. As a result, the crosslinked PVA nanofibers adhered to each other and the fibrous structure partially dissolved, resulting in a dense film after being immersed in water for 120 min (Figure S1 in Supplementary Materials). When the processing temperature was 160 °C, the crosslinked PVA/EH/CBS nanofibers were able to maintain their fibrous morphology in water. When the heating temperature reached 180 °C, PVA nanofibers began to turn yellow and became brittle, and in the meantime, their mechanical strength decreased apparently. The weight loss of PVA/EH/CBS nanofibers in water was only 10.57% at 160 °C, and the further increase in temperature made hardly any improvement on their water resistance; therefore, the temperature of 160 °C was applied as the heating temperature in this research.

When the heat treatment time was less than 10 min, the PVA/EH/CBS nanofiber membranes lost their fiber contour with enclosed pores after being immersed in water. When the treatment temperature was longer than 10 min, the fiber morphology could be well-conserved after being soaked in water as shown in Figure S2. The mass loss results in Figure 3b coincided with the above results that the mass loss changes did not decrease from 10 min heating treatment. The prolonged treatment did not further improve the fiber stability in water but led to declined mechanical strength; therefore, the heat treatment time was set as 10 min. We also explored the influence of PVA/EH mass ratio on the water resistance of nanofibers, while the Na_2_CO_3_/NaHCO_3_ molecular ratio was set as 3:2. The prepared nanofibers with different EH contents were treated at 160 °C for 10 min, and they had smaller weight loss in water with the growing EH content, as expected (Figure 3c). 

The addition of CBS in the electrospinning solution can adjusted not only the pH value, but also affected electrical conductivity of solutions and electrospinning performances accordingly. Figure 3d shows that the pH of PVA/EH/CBS solutions declined with the reducing Na_2_CO_3_/NaHCO_3_ molar ratio. Na_2_CO_3_ and NaHCO_3_ are strong electrolytes that completely ionized in the aqueous solution, CO_3_^2^^−^ had secondary hydrolysis and HCO_3_^−^ could only be partially hydrolyzed. As the CO_3_^2^^−^ mass ratio reduced, the solution pH decreased accordingly. The molar ratio of Na_2_CO_3_/NaHCO_3_ had an insignificant effect on the conductivity of solutions as shown in Figure 3e, probably because their total molar ratio remained unchanged and the amount of PVA and EH played a decisive role in the solution conductivity. The addition of CBS enhanced the electrostatic force applied to the solution jet, resulting in improved jet velocity and amount of solution ejected, which in turn led to increased fiber diameter, from 303.4 nm to 316.6 nm.

Figure 3f shows the fiber diameter difference of PVA/EH/CBS nanofibers before and after immersion in water, and the small difference revealed improved crosslinking density, with the morphology of corresponding nanofibers shown in Figure S3. Because HCO_3_^−^ is less basic with less catalytic effect than CO_3_^2^^−^, the obtained nanofibers at smaller Na_2_CO_3_/NaHCO_3_ molar ratios had larger swelling ratios, showing lower water resistance. When the molar ratio of Na_2_CO_3_/NaHCO_3_ was 0:5, the obtained nanofibers adhered together forming a highly interconnected fibrous structure. On the contrary, PVA/EH/CBS nanofibers at high Na_2_CO_3_/NaHCO_3_ molar ratios showed high stability and small diameter change in water. However, when the Na_2_CO_3_/NaHCO_3_ molar ratio was 4:1 and higher, the solution had strong alkalinity, and epoxy groups on EH partially hydrolyzed resulting in a loss of reactivity [40] and deteriorated crosslinking efficiency. Even electrospinning failed when the Na_2_CO_3_/NaHCO_3_ molar ratio was 5:0. It can be concluded from the results that the application of CBS can facilitate epoxy ring opening and catalyze etherification reaction between epoxy and hydroxyl groups. PVA/EH/CBS nanofibers had the smallest fiber diameter variation after being immersed in water and the best water resistance when the molar ratio of Na_2_CO_3_/NaHCO_3_ was 3:2.

**Figure 4:**
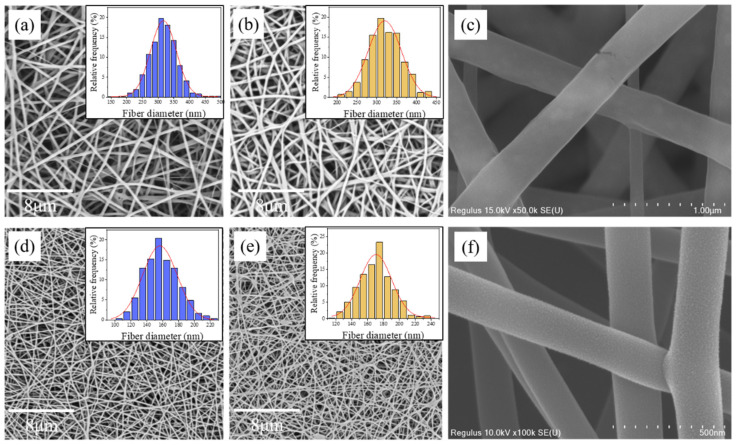
SEM images of PVA/EH/CBS nanofibers (**a**) before immersion in water, (**b**) immersion in water, (**c**) enlarged view of fibers and PVA/BI nanofibers (**d**) before immersion in water, (**e**) immersion in water, (**f**) enlarged view of fibers.

Each structural unit of PVA contains one hydroxyl group, and these hydroxyl groups can form intramolecular and intermolecular hydrogen bonds that reduce the mean square end distance between PVA molecules and form restricted random coils [42]. The interaction of intermolecular hydrogen bonds could form cohesive entanglement points among molecule chains leading to a random coil aggregate structure in the PVA solution. This structure may inhibit the crosslinking agents from contacting hydroxyl groups on PVA molecule chains and reduce the crosslinking efficiency. Therefore, the breakdown of PVA aggregate structure in the solution could enable hydroxyl and epoxy groups to have more chances to collide and react, as a result, increasing the crosslinking efficiency. To prove the above speculation, we used water-based blocked isocyanate (BI) to crosslink PVA nanofibers, which also had high reactivity with hydroxyl groups. Figure 4 shows PVA/BI nanofibers also had a uniform fiber morphology, and both water-soluble crosslinking agents had excellent crosslinking performances as the obtained nanofibers had hardly any morphology change after immersion in water. PVA/EH/CBS nanofibers had an average diameter of 316.6 nm, which grew to 321.7 nm after immersion in water with only a 1.6% increase. The average diameter of the PVA/BI nanofibers was 158.0 nm, which increased to 167.1 nm after immersion in water, an increase of 5.8%, indicating less stability of PVA/BI nanofibers. It can be clearly seen from the enlarged views of nanofibers (Figure 4c,f) that PVA/EH/CBS had a smooth fiber surface and PVA/BI nanofibers had a rough surface with grain structure on the surface. The distinctive grains on the surface of PVA/BI nanofibers were concentrated on the fiber surface, and these grains may belong to BI.

**Figure 5:**
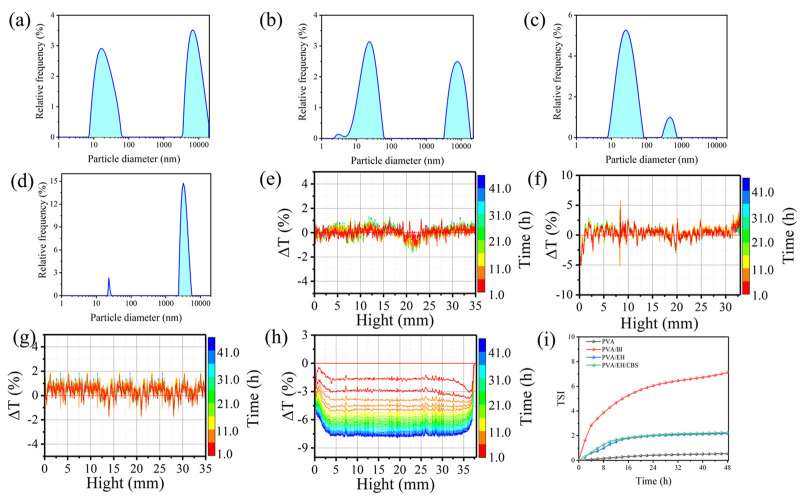
Particle size distribution of of (**a**) PVA solution, (**b**) PVA/EH solution, (**c**) PVA/EH/CBS solution (Na_2_CO_3_/NaHCO_3_ molecular ratio = 3:2), (**d**) PVA/BI dispersion and dispersion of the transmitted light scattering curves of (**e**) PVA, (**f**) PVA/EH, (**g**) PVA/EH/CBS, (**h**) PVA/BI, (**i**) TSI change with time (solution concentration 80 g/L and PVA to crosslinking agent mass ratio 1:1).

To validate our postulation that BI do not evenly distribute in the nanofibers, the particle size distribution of PVA, PVA/EH, PVA/EH/CBS solutions and PVA/BI dispersion were measured by the laser particle size analyzer as shown in Figure 5a–d. The PVA solution had two peaks, the peak at 20–100 nm was composed of free PVA molecule chains isolated from each other and the intramolecular hydrogen bonds mainly existed between molecule chains. The peaks in the range of 6000–20,000 nm were composed of the topological entanglement structure caused by the mutual hooking of PVA macromolecule chains and the cohesive entanglement structure induced by the intermolecular hydrogen bonds. The addition of EH had no significant influence on the particle size of PVA solution, albeit, there was a new peak located at 3.03 nm attributed to the formation of EH molecule clusters in the solution. Figure 5c shows that CBS can break the large aggregate structure of PVA into small ones, with the average particle size of 400 nm. This is because the PVA/EH/CBS solution is alkaline and O^2^^−^ in OH^−^ has strong electron donating ability which can weaken hydrogen bonds between PVA molecules and break the entanglement of molecule chains as shown in Figure 1. It is also interesting to notice that CBS can also break down the EH molecule clusters and improve their dispersion in the solution, as evidenced by the disappearance of the peak at 3.03 nm. 

The molar ratio of CBS also had an effect on the particle size of PVA solutions, and the strong alkaline solution was more efficient in breaking PVA particles (shown in Figure S4). The PVA/EH/CBS solutions with larger CBS ratios (4:1, 3:2, 2:3) could easily break PVA particles into small ones, whereas those with smaller ratios (1:4 and 0:5) had difficulty in breaking down all the large aggregate structures, with large particle peaks still shown at 7600 nm and 13,000 nm. Therefore, EH molecules uniformly dispersed and PVA did not form a large coil aggregate structure in the PVA/EH/CBS solution. When BI was added to the PVA solution, the BI emulsion could interact with isolated PVA molecules and PVA molecule aggregate, and as a result, the particle size in the PVA/BI dispersion shifted to 3500 nm, except that there was a peak at 20–100 nm assigned to isolated PVA molecule chains in the solution. It can be concluded that the BI was in an emulsion form, and it was not mixed homogeneously with PVA molecules, resulting in a PVA-rich phase and a BI-rich phase, which verified the previous SEM results. The rough morphology of PVA/BI nanofibers could be the outcome of solidified BI emulsion and large PVA molecule aggregate migrated to the fiber surface.

The stability of solutions is also investigated by monitoring the changes in transmission (T) and backscatter (BS) signals of the solutions as shown in Figure 5e–h. PVA, PVA/BI, PVA/EH and PVA/EH/CBS solutions were stable without showing apparent change in the ΔT value, and the PVA/BI dispersion had dramatic change in the ΔT value indicating low solution stability. The solution stability was also evaluated by calculating the TSI (Turbiscan stability index) value:

(2) $$\mathrm{TSI}=\frac{\sum_{h}│S_{i}\left(h\right)-S_{i-1}\left(h\right)│}{H}$$

where S_i_(h) is the average value of backscattering light intensities at a height of h, S_i−1_(h) is the backscattering light intensity of the previous scan and H is the height of sample in the glass cylinder sample bottle. In general, the TSI value of a stable solution is not affected by the rest time. Figure 5i shows that the PVA and PVA/EH solutions had low TSI values, indicating they are stable with time. The TSI curve of PVA/BI dispersion had a steep slope with higher values than PVA/EH/CBS solutions, revealing that the PVA/BI dispersion was not stable which was consistent with the transmitted light intensity results. This is because the BI emulsion in the PVA solution collided with PVA molecules continuously and eventually caused agglomeration. 

**Figure 6:**
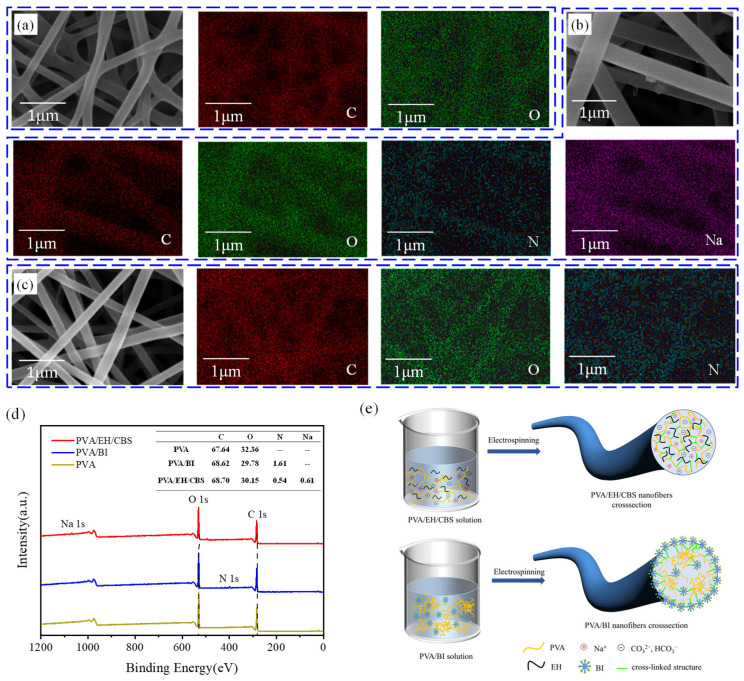
EDS mapping spectra of elements C, O, N and Na on (**a**) PVA, (**b**) PVA/EH/CBS and (**c**) PVA/BI nanofibers, (**d**) XPS spectra of PVA, PVA/EH/CBS and PVA/BI nanofibers, (**e**) Schematic illustration of distribution of PVA/EH/CBS and PVA/BI in the dispersions.

The distribution of PVA and crosslinking agents in the nanofibers was investigated by EDS and XPS. The molecular formula of EH was C_12_H_24_O_2_N_2_ and the molecular formula of BI was C_22_H_34_O_6_N_4_. The EDS mapping results in Figure 6a–c confirmed crosslinking agents had been introduced into nanofibers. Since the detection depth of XPS is around 10 nm smaller than the nanofiber diameter, the XPS results only revealed the composition of fiber surfaces. When the amount of EH and BI added is the same, the molar ratio of N in PVA/EH/CBS and PVA/EH solution should be 1:1.07. Figure 6d shows that the N molar ratio of PVA/EH/CBS and PVA/EH nanofibers was 1:2.98, which is much larger than the theoretical value. Therefore, it can be concluded that BI was not uniformly dispersed inside the nanofibers but more accumulated on the fiber surface. 

XPS spectra of C1s and O1s of PVA/EH/CBS nanofibers are provided in Figure 5, and there were three independent peaks at 284.80, 286.22 and 289.29 eV, respectively, attributed to C−C/C−H (carbon skeleton of PVA), C−O/C−O−C (epoxy and hydroxyl groups) and C=O/O−C=O (ester groups) [43]. The two independent peaks of 530.16 eV and 531.14 eV were attributed to the C=O and hydroxyl (O−H) on the ester groups on the PVA molecular chains [43]. In addition, the C=O content was significantly higher than that of O–H. This is because hydrophobic ester groups are prone to distribute at the gas–liquid interface to reduce surface energy during the electrospinning process. The high-resolution results of C1s and O1s of PVA/BI nanofibers were similar to those of PVA/EH/CBS nanofibers.

Combing the particle size results and XPS results of nanofibers, it seems reasonable to postulate that the homogeneous distribution of EH in the solution breaks down PVA coil aggregates, and as a result, EH distributed uniformly within the nanofibers which enabled hydroxyl and epoxy groups to have a large chance to contact for esterification reaction as shown in Figure 6e. The uniform distribution of crosslinking agent was proved to improve the crosslinking efficiency and water resistance of stabilized PVA nanofibers. As for the PVA/BI dispersion, BI emulsion was not homogeneously dispersed in the dispersion resulting in the existence of a BI-rich phase and PVA aggregate structures, which led to the rough surface of PVA/BI nanofibers. Figure 4f and Figure 5h prove the above point. The aggregate structure of PVA molecule chains can inhibit the contact between hydroxyl and isocyanate groups and affect the crosslinking efficiency.

## 4. Conclusions

Water-soluble epoxy crosslinking agent EH was applied to stabilize electrospun PVA nanofibers. The addition of CBS significantly improved the crosslinking reaction. When the heating temperature was 160 °C, the water resistance of the crosslinked PVA nanofibers reached the best result after heat treatment for 10 min. FTIR spectroscopy confirmed that the hydroxyl groups of PVA and epoxy groups of EH formed chemical crosslinks. The particle size results showed that the particle size of the PVA molecule chains could be effectively reduced from 8335.2 nm to 467.2 nm and contribute to the formation of stable electrospinning solutions. The use of an epoxy EH/CBS system that uniformly disperses in PVA solutions can significantly improve the crosslinking degree and the water resistance of PVA nanofibers. For comparison, water-based blocked isocyanate was applied to crosslink PVA nanofibers, with lower water stability due to the phase separation of PVA/BI dispersion. This work provides a new strategy to develop high efficiency water-based crosslinking agents for stabilizing electrospun nanofibers.

## Figures and Tables

**Table 1:** Concentrations of sodium carbonate and sodium bicarbonate in the solution.

Serial Number	Na_2_CO_3_ (mol/L)	NaHCO_3_ (mol/L)	Na_2_CO_3_/NaHCO_3_ Molar Ratio
1	0.2	0	5/0
2	0.16	0.04	1/4
3	0.12	0.08	3/2
4	0.08	0.12	2/3
5	0.04	0.16	1/4
6	0	0.2	0/5

## Data Availability

Data is available on request.

## Supplementary Materials

The following supporting information can be downloaded at: https://www.mdpi.com/article/10.3390/molecules27134177/s1, Figure S1: SEM images after immersion in water at different heating temperatures; Figure S2: SEM images after immersion in water for different heating times; Figure S3: SEM image of nanofibers immersed in water after electrospinning in different molar ratios of sodium carbonate/sodium bicarbonate buffer solutions; Figure S4: Particle size distribution of different ratios of Na_2_CO_3_/NaHCO_3_ in polyvinyl alcohol solution; Figure S5: High resolution XPS spectra.

## Sample Availability

Samples of the compounds are available from the authors.
