# Supplementary material for: A Water-Soluble Epoxy-Based Green Crosslinking System for Stabilizing PVA Nanofibers
Source: Molecules. 2022 Jun 29;27(13):4177. doi: 10.3390/molecules27134177 (PMC9267985; doi:10.3390/molecules27134177)
Supplement: Supplementary file 1 [file molecules-27-04177-s001.zip › molecules-1792766-supplementary.pdf]

molecules

MDPI

Supplementary Material

A Water-Soluble Epoxy Based Green Crosslinking System for Stabilizing PVA Nanofibers

Yujian Zhang [1], Kuanjun Fang [1,*], Wei Wang [1] and Haitao Niu [1,2,*]

1 College of Textiles & Clothing, Qingdao University/State Key Laboratory for Biofibers and Eco-textiles/Collaborative Innovation Centre for Eco-textiles of Shandong Province, 308 Ningxia Road, Qingdao 266071, China. jadechang@163.com(Y.Z.);13808980221@163.com(K.F.); 1159510182@qq.com(W.W.); niuhaiao78@163.com(H.N.)
2 Jiangsu New Vision Advanced Functional Fiber Innovation Center, Shengze Town, Wujiang City, Jiangsu Province, 215228, China. niuhaitao78@163.com(H.N.)
* Correspondence: 13808980221@163.com(K.F.); niuhai-tao78@163.com(H.N.)

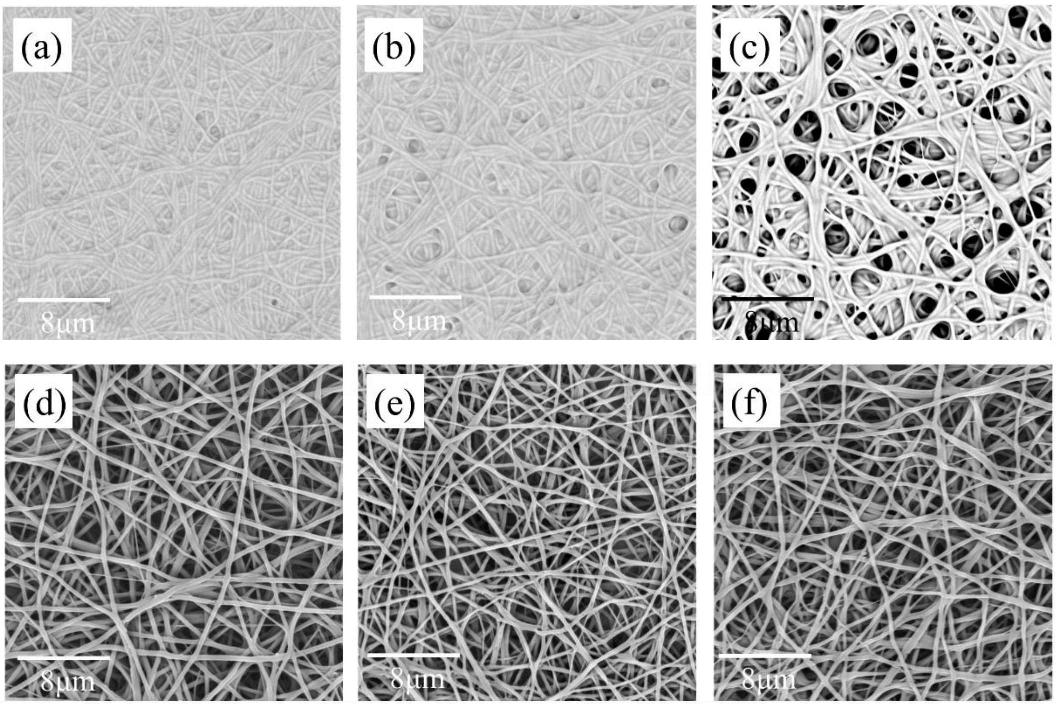

Figure S1. SEM images after immersion in water at different heating temperatures (**a**)100℃, (**b**)120 ℃, (**c**)140℃, (**d**)160℃, (**e**)180℃, (**f**)200℃.

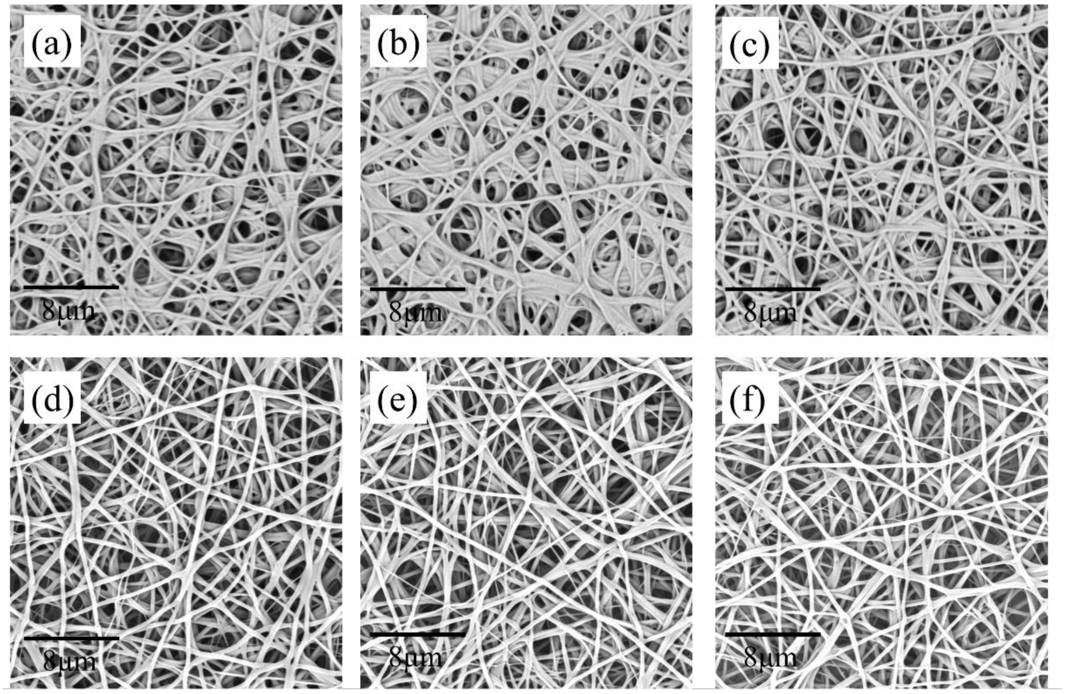

Figure S2. SEM images after immersion in water for different heating times(**a**)1min, (**b**)3min, (**c**)5min, (**d**)10min, (**e**)15min, (**f**)30min.

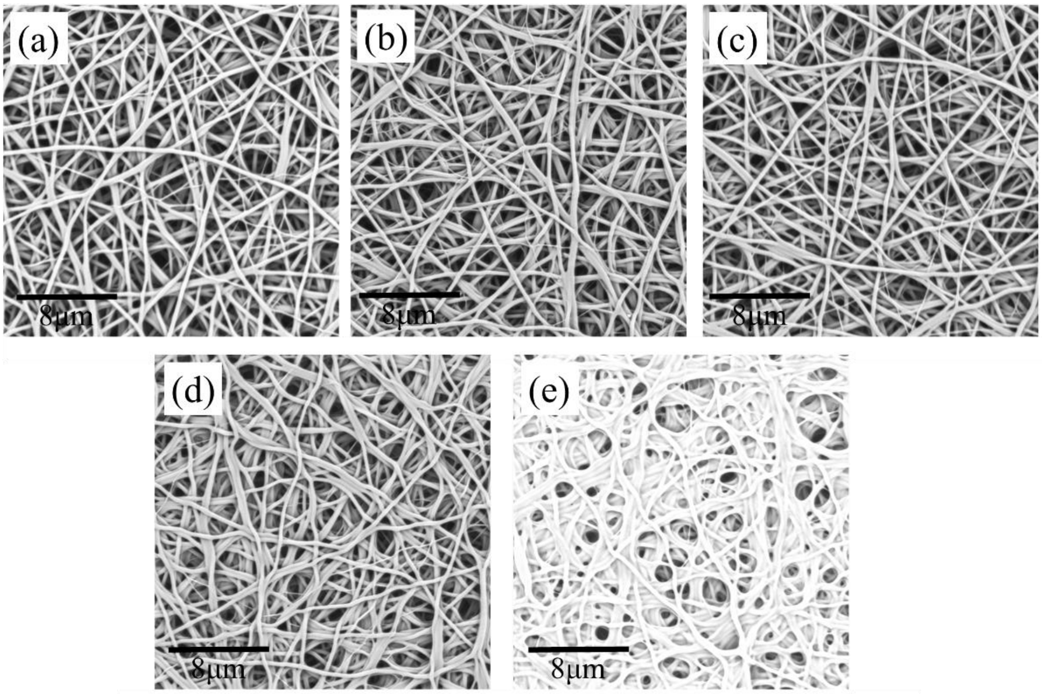

Figure S3. SEM image of nanofibers immersed in water after electrospinning in different molar ratios of sodium carbonate/sodium bicarbonate buffer solutions (**a**)4:1, (**b**)3:2, (**c**)2:3, (**d**)1:4, (**e**)0:5.

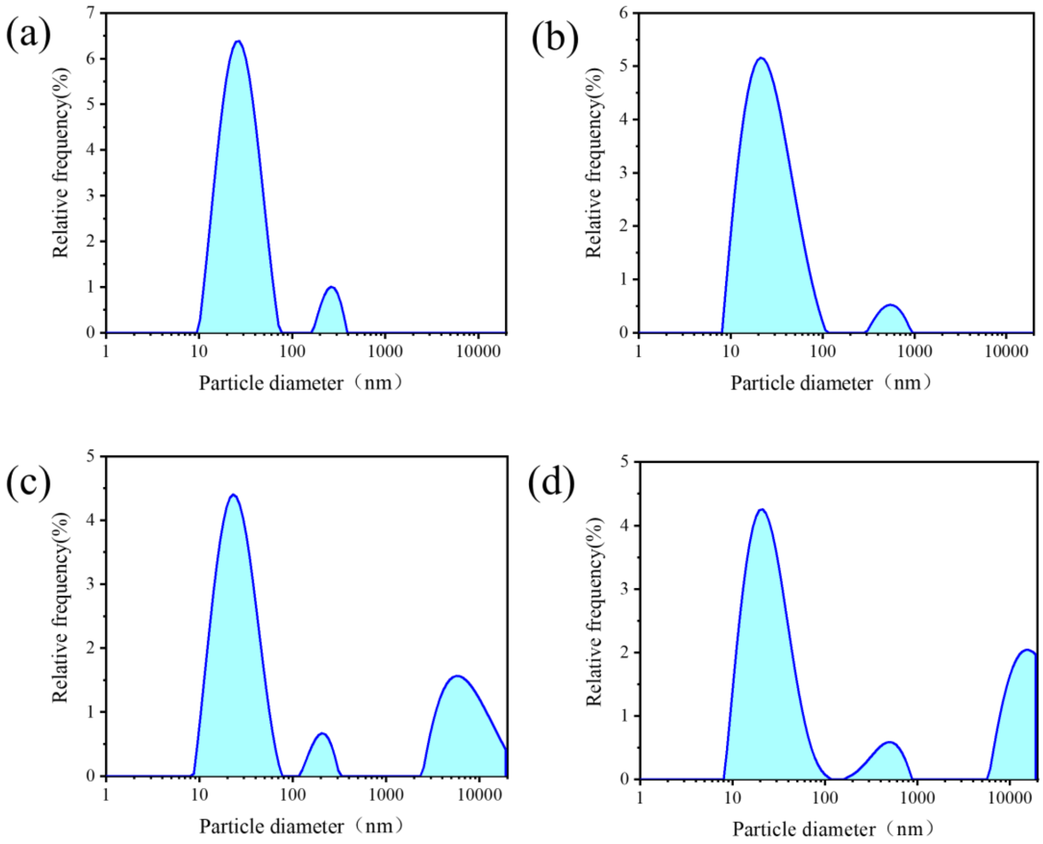

Figure S4. Particle size distribution of different ratios of $Na_2CO_3/NaHCO_3$ in polyvinyl alcohol solution (**a**)4:1, (**b**)2:3, (**c**)1:4, (**d**)0:5.

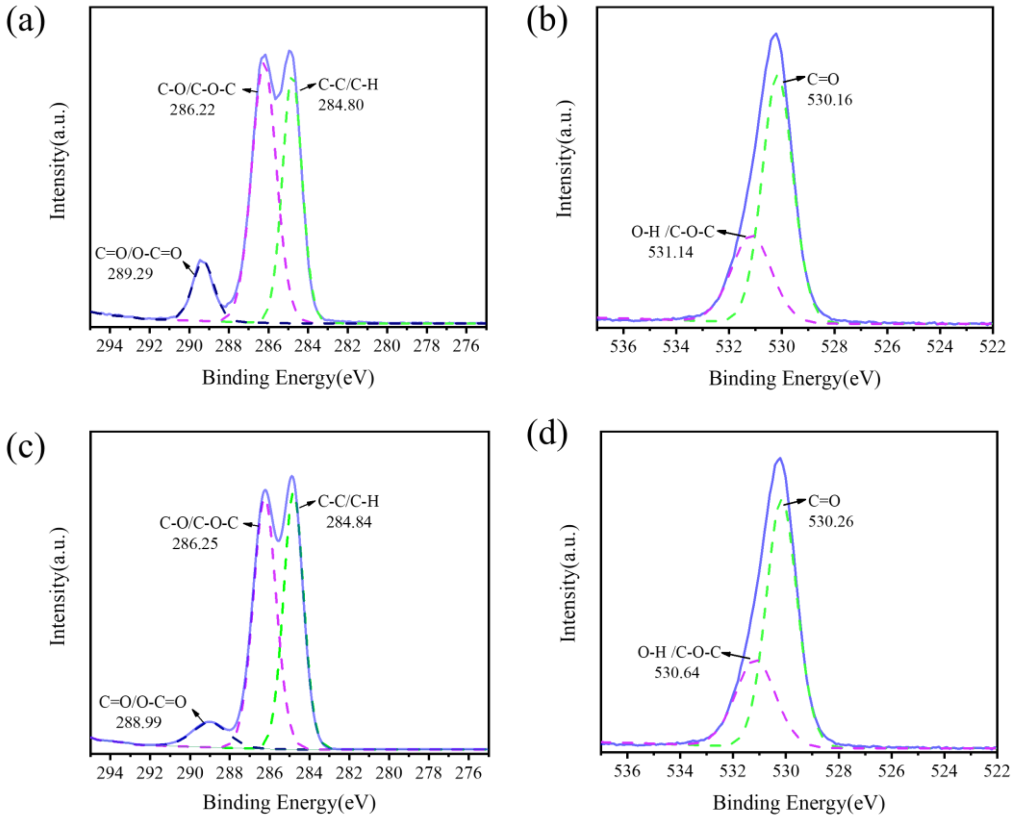

Figure S5. High resolution XPS spectra (**a**) PVA/EH/CBS C1s, (**b**) PVA/EH/CBS O1s, (**c**)PVA/BI C1s, (**d**) PVA/BI O1s.
